# Risk prediction models for postmenopausal osteoporosis: a systematic review and meta-analysis study

**DOI:** 10.1186/s12891-025-09385-2

**Published:** 2026-01-08

**Authors:** Lingjia Li, Xiangzhou Lan, Weike Zeng, Yujiao Xu, Qing Chen

**Affiliations:** 1 The first hospital of Hunan university of traditional Chinese medicine, Changsha, Hunan Province China; 2 Hunan university of Chinese medicine, Changsha, Hunan Province China; 3 Changsha contemporary nurses magazine limited, Changsha, Hunan Province China; 4 The second affiliated hospital of Soochow university, Suzhou, Jiangsu Province China

**Keywords:** Machine learning, Postmenopausal osteoporosis, Systematic review

## Abstract

**Background:**

Postmenopausal osteoporosis usually happens 5 ~ 10 years after menopause. Low awareness, low detection rates and high morbidity have prevented the possibility of early or preventive interventions, thus increasing the social and economic burden on families and societies. A reliable prediction model for postmenopausal osteoporosis has the potential to guide the prevention, but regarding the early prediction of postmenopausal osteoporosis without fracture, this field has not been sufficiently studied. Although many scholars have developed several prediction models to estimate the risk of postmenopausal osteoporosis without fractures, the evidence about the model quality and clinical applicability is scarce.

**Method:**

Nine databases (Medline, Embase, Web of science, CINAHL, The Cochrane Library, CNKI, SinoMed, Wanfang, VIP data) were systematically searched from 1 January 2014 to 1 May 2024. Two researchers independently extracted data using the CHARMS checklist and assessed bias using the PROBAST tool. The primary outcomes of interest were related to the model’s discriminative ability (assessed by pooled AUC values) and calibration performance (evaluated using calibration curves or the calibration intercept and slope). We performed meta-regression and sensitivity analyses to explore the influence of important factors, such as data sources, machine learning methods, and types of predictor variables, on the aforementioned. results. Additionally, subgroup analyses were conducted based on data sources, machine learning. methods, and types of predictor variables. The study was registered in the PROSPERO database (registration number CRD42024542498).

**Results:**

A total of 8,549 records were initially identified, and 7 studies (comprising 19 models) were ultimately included. All models were developed based on Asian population data. The risk of bias assessment showed: 1 study had a low risk, 1 study had an unclear risk, and 5 studies had a high risk. The sample sizes ranged from 319 to 4,417 participants. The reported AUC of the models ranged from 0.639 to 0.921; however, the vast majority of studies lacked reports on calibration performance. The pooled C-statistic (AUC) was 0.78 (95%CI: 0.73–0.83). Sensitivity analysis yielded robust results (AUC=0.77). Subgroup analysis indicated that models combining demographic and laboratory data demonstrated the best performance (AUC=0.92). Significant publication bias and substantial heterogeneity (I² = 98%) were observed among the studies.

**Conclusion:**

Current machine learning-based prediction models for postmenopausal osteoporosis without fractures, as presented in the included studies, demonstrate good discriminative ability but are generally characterized by a high risk of bias, a notable lack of calibration performance evaluation, and insufficient validation of clinical utility. Furthermore, existing models are developed entirely on Asian population data, which limits their generalizability to other populations. Future research should focus on strictly adhering to prediction model research guidelines (such as PROBAST), enhancing the reporting of model calibration and clinical utility, and assessing model generalizability through external validation in multi-center studies encompassing diverse ethnicities and regions.

**Supplementary Information:**

The online version contains supplementary material available at 10.1186/s12891-025-09385-2.

## Introduction

Osteoporosis (OP) is a metabolic bone disease characterized by reduced bone density, degeneration of the trabecular structure and increased risk of fractures. Osteoporosis-related fractures impose a substantial socioeconomic burden worldwide. Annual expenditures reach approximately $1.79 billion in the United States and £4 billion in the United Kingdom [1]. Globally, the prevalence of osteoporosis remains alarmingly high among women, with an estimated rate of 23.1%. Postmenopausal women are particularly vulnerable due to a dramatic decline in estrogen levels caused by ovarian function deterioration, which significantly disrupts bone metabolism and places them in the highest-risk category [2]. Postmenopausal osteoporosis (PMOP) affects approximately 200 million women worldwide, with significant regional variations in prevalence. Morocco reports a pooled prevalence rate of 32% [3]. In Asia, particularly in populous countries like China, the prevalence of OP among women aged 50 and above has been reported to be significantly higher than in some European and American countries [4]. However, a key global challenge remains the widespread lack of awareness about PMOP among patients. Regional surveys indicate that in some Asian countries, the awareness rate of OP among women aged 40–49 is as low as 0.9% [5], although more comprehensive and updated data are still needed. Early prevention and timely diagnosis are crucial for maintaining bone mineral density and slowing disease progression. According to the World Health Organization (WHO) diagnostic criteria for osteoporosis [6], the bone density measured compared to the peak bone density of the same sex, standard deviation of the bone density decreased (BMD) by T value ≤ −2.5 can be diagnosed as osteoporosis. However, the accessibility and utilization of BMD testing are generally inadequate. Studies have shown that the rate of BMD testing among postmenopausal women after sustaining a distal radius fracture remains low and has shown a declining trend, once being as low as 10.5% [7]. This issue is even more severe in the general population and in resource-limited settings (e.g., reports indicate that the testing rate in China was once as low as 3.7%) [5]. Low awareness, low detection and high prevalence have resulted in most patients not taking timely preventive and control measures in the early stages of bone loss until pain, spinal deformation and fractures occurring, which prevents the possibility of early or preventive interventions, thus increasing the social and economic burden on families and societies. A range of prescreening tools are developed to predict the risk of osteoporosis, such as the osteoporosis self-assessment tool for Asians [8], the osteoporosis risk assessment instrument [9], simple calculated osteoporosis risk estimation [10] and so on. However, these tools exhibit significant limitations in predictive performance due to their oversimplification of the complex pathophysiology of bone metabolism. Their area under the receiver operating characteristic curve (AUROC) typically ranges between 0.65 and 0.75 [11–13], which substantially restricts their application in high-precision risk stratification and poor predictive performance leads to missed opportunities for early intervention. Moreover, they demonstrate limited capacity to effectively incorporate the increasingly available clinical data, particularly serum biochemical markers that are closely associated with bone metabolism. 

 In recent years, with the accumulation of medical big data and advancements in artificial intelligence, machine learning (ML) technology has demonstrated strong potential in medical prediction fields. The core advantage of ML models lies in their ability to learn nonlinear relationships and complex interactions between features, potentially overcoming the limitations of traditional tools. By integrating multidimensional data including demographic information, clinical variables, and key serum bone metabolism markers (such as bone turnover markers, vitamin D, and hormone levels), machine learning models provide a new opportunity to develop more accurate risk prediction tools for the PMOP population without fractures. However, current research still exhibits significant gaps in external validation and clinical generalizability of these models. Tartibian B et al. [14] employed the k-nearest neighbors (KNN) algorithm to predict osteoporosis risk in elderly women, achieving 61.7% accuracy in identifying femoral neck OP risk. The study proposed this method could serve as a preliminary screening tool prior to DXA scans, thereby reducing unnecessary testing. Subsequently, Fasihi L et al. [15] systematically compared eight algorithms and demonstrated that gradient boosting (GB) and random forest (RF) outperformed KNN for OP prediction, while Ada Boost proved most suitable for generating exercise prescriptions. Notably, the study developed an innovative “prediction-intervention” closed-loop system that directly outputs exercise protocols (e.g., recommending aquatic training combined with balance exercises for osteoporotic women), effectively shortening clinical decision-making pathways and addressing personalized healthcare needs. It must be pointed out that the safety and appropriateness of personalized exercise programs generated by such models still require evaluation and confirmation by trained exercise or medical professionals. These studies demonstrate that ML models exhibit good discriminative ability for OP risk prediction and possess potential as clinical decision-support tools.

Simultaneously, the development of OP risk prediction models faces significant challenges. Wu et al. [16] evaluated 53 osteoporosis prediction models involving 15,209,268 patients. Their study revealed that while machine learning demonstrated good performance in fracture prediction (with relatively high pooled C-indices), the vast majority of models lacked adequate external validation to confirm their reliability and generalizability. This insufficient validation landscape substantially restricts the reliable application of models across diverse populations and clinical settings, constituting a critical bottleneck hindering the clinical translation of ML technologies. The development of rigorously externally validated PMOP-specific prediction models with robust generalizability has thus emerged as a pivotal scientific challenge in the field. Compared to the study by Wu Yu et al., which primarily evaluated fracture risk prediction in diagnosed patients, the core innovation of this research lies in its precise focus on the early risk prediction of PMOP without fracture, which is a critical yet under explored area of study.

Therefore, this study aims to systematically evaluate the current research status of machine learning models in predicting the risk of PMOP without fracture. The study will emphasize analyzing the predictive performance of existing models, rigorously assessing their external validation status and evidence of clinical generalizability, and systematically introducing a comprehensive review of model reporting quality and methodological bias risks. Through this systematic evaluation, the study aims to provide evidence-based insights and optimization strategies for developing more robust and clinically translatable risk prediction tools of PMOP without fracture, thereby facilitating early identification and intervention in high-risk populations and ultimately reducing the incidence and disease burden of this major public health issue.

## Method

This systematic review and meta-analysis were according to the preferred reporting items suggested by the Cochrane Collaboration. The meta-analysis was registered in the PROSPERO database (registration number CRD42024542498). The study protocol explicitly defined the research questions, search strategy, inclusion/exclusion criteria, data extraction items, risk of bias assessment methods, and planned analytical approaches. The final version was publicly available in the PROSPERO registry record or could be obtained by contacting the corresponding author.

### Search strategy

We conducted a systematic search of the literature from Medline, Embase, Web of science, CINAHL, The Cochrane Library, CNKI, SinoMed, Wanfang, VIP data. Search time ranged from 1 January 2014 to 1 May 2024. Articles were searched in databases by combining related terms such as “postmenopausal osteoporosis”, “bone loss”, “machine learning”, “prediction model”. We applied a prediction model-specific filter (Hayden filter [17]) and manually traced reference lists. The complete search strategy is available in Supplementary File 1 (S1 search strategy and S2 study selection criteria). As this study was based on previously published research, it was not applicable for permissible consent from participants.

## Inclusion and exclusion criteria

### Inclusion criteria as follows

This study established the inclusion criteria based on the PICOS principle, as follows: (1)Population (P): Postmenopausal women. (2)Study Design (I): Cohort studies or case-control studies that involved the development and/or validation of prediction models for postmenopausal osteoporosis without fractures. (3)Prediction Model (C): The core of the study must be the development, validation, or updating of a prediction model. (4)Outcome (O): The primary outcome is the diagnosis of postmenopausal osteoporosis (based on a bone density T-score ≤ −2.5 as measured by DXA. (5) Model Performance (S): The study must report at least one model performance metric.

## Exclusion criteria as follows

(1) the content of the study only referred to predictors or risk factors, no predictive model had been established; (2) used qualitative methods to construct prediction models; (3) reported repeatedly, abstracts, conference papers, cases, and reviews; (4) Non-Chinese and English.

## Data extraction

Two researchers (L.J.L. and X.Z.L.) independently extracted the data from the included studies. For data discrepancies, two independent reviewers will first resolve disagreements through discussion. If consensus cannot be reached, a third senior investigator (Y.J.X.) will arbitrate the final decision. For studies with missing original data, we tried to contact the author to request the data. If no response was received, we would attempt to derive the required data through reported results (e.g., calculating standard errors from confidence intervals [18]). Studies would only be excluded when key performance metrics were completely unavailable and cannot be reasonably estimated, with all exclusion reasons being systematically documented. For studies reporting multiple models within the same publication, we extracted performance data from all eligible models reported in that study. For instances where multiple versions of the same model were reported across different publications, we prioritized the inclusion of the final version, the one with the most complete set of predictors and the most thorough validation to avoid duplicate counting of the same model population. The information extracted was according to the checklist for critical appraisal and data extraction for systematic reviews of prediction modeling studies (CHARMS) [19]. The details were as follows: (1) Basic study information: first author, publication year, country, data source. (2) Study population and outcome: sample size, number of osteoporosis events, sample size adequacy (the events per variable, EPV) [20]. (3) Predictors: list of candidate predictors, predictors finally included in the model, type of predictors (demographic characteristics primarily including age, BMI, age at menopause, height, etc., laboratory indicators primarily biochemical and serum markers, other indicators primarily lifestyle factors, medication use, and nutritional intake). (4) Model development methods: predictor selection method, method for handling missing data, model algorithm (e.g., Logistic Regression, RF, etc.), type of validation. (5) Model performance metrics: ①Discrimination: Refers to the model’s ability to distinguish between individuals with and without the disease. Extracted metrics include the C-statistic/area under the receiver operating characteristic curve (AUC), sensitivity, specificity, etc. ② Calibration: Refers to the agreement between the predicted risk by the model and the actual observed risk. Extracted metrics include p-value of the Hosmer-Lemeshow test, calibration slope, etc. ③ Other metrics: accuracy, positive predictive value (PPV), negative predictive value (NPV), and Youden’s index were also extracted. ④ Clinical utility: Record whether decision curve analysis (DCA) was reported to evaluate the model’s net clinical benefit. (6) Model presentation: Extract the final presentation format of the model (e.g., nomogram, online calculator, etc.).

### Risk of bias assessment

Prediction model risk of bias assessment tool (PROBAST) [21] was used to assess the bias risk of models. Two reviewers (L.J.L. and X.Z.L.) used the tool to assess each article independently from four domains: study subjects, predictors, outcomes and statistical analysis. During assessment, each question was answered as yes, probably yes, probably no, no, or no information, with yes indicating a low risk of bias and no indicating a high risk of bias. After completing independent assessments, the reviewers would enter their results into standardized forms for comparison. For any discrepancies identified, the two reviewers would first discuss and attempt to reach consensus. If disagreements persist after discussion, a third senior investigator (Y.J.X.) would review the relevant evidence and make the final determination.

### Data analysis

We used a random effects model to pool AUCs across studies and presented the results in forest plots. The degree of inter-study heterogeneity using the Cochran’s Q test [22] (where significant heterogeneity was defined as *P* ≤ 0.10 or *I²* > 50%) to assess whether a fixed effects model could have been used. We conducted meta-regression analyses to investigate potential sources of heterogeneity and performed subgroup analyses to examine the consistency of effects based on data sources (database vs. electronic medical record), machine learning methods (LR vs. others), and types of predictors (demographics vs. demographics and other indicators vs. demographics, laboratory and others), with a predefined significance threshold of *P* < 0.05. Sensitivity analysis was performed to further identify the source of heterogeneity by removing each study and re-calculating the pooled effect size of the remaining studies. Publication bias was assessed using Egger’s linear regression test, with a significance threshold of *P* < 0.05 indicating potential bias. In cases of funnel plot asymmetry, the trim-and-fill method was applied to adjust for potential missing studies. The meta analysis was performed using the software R V.4.2.2 (R Development Core Team, Vienna, http://www.R-project.org). It was considered statistically significant that p value less than 0.05.

## Results

### Study selection

**Fig. 1:**
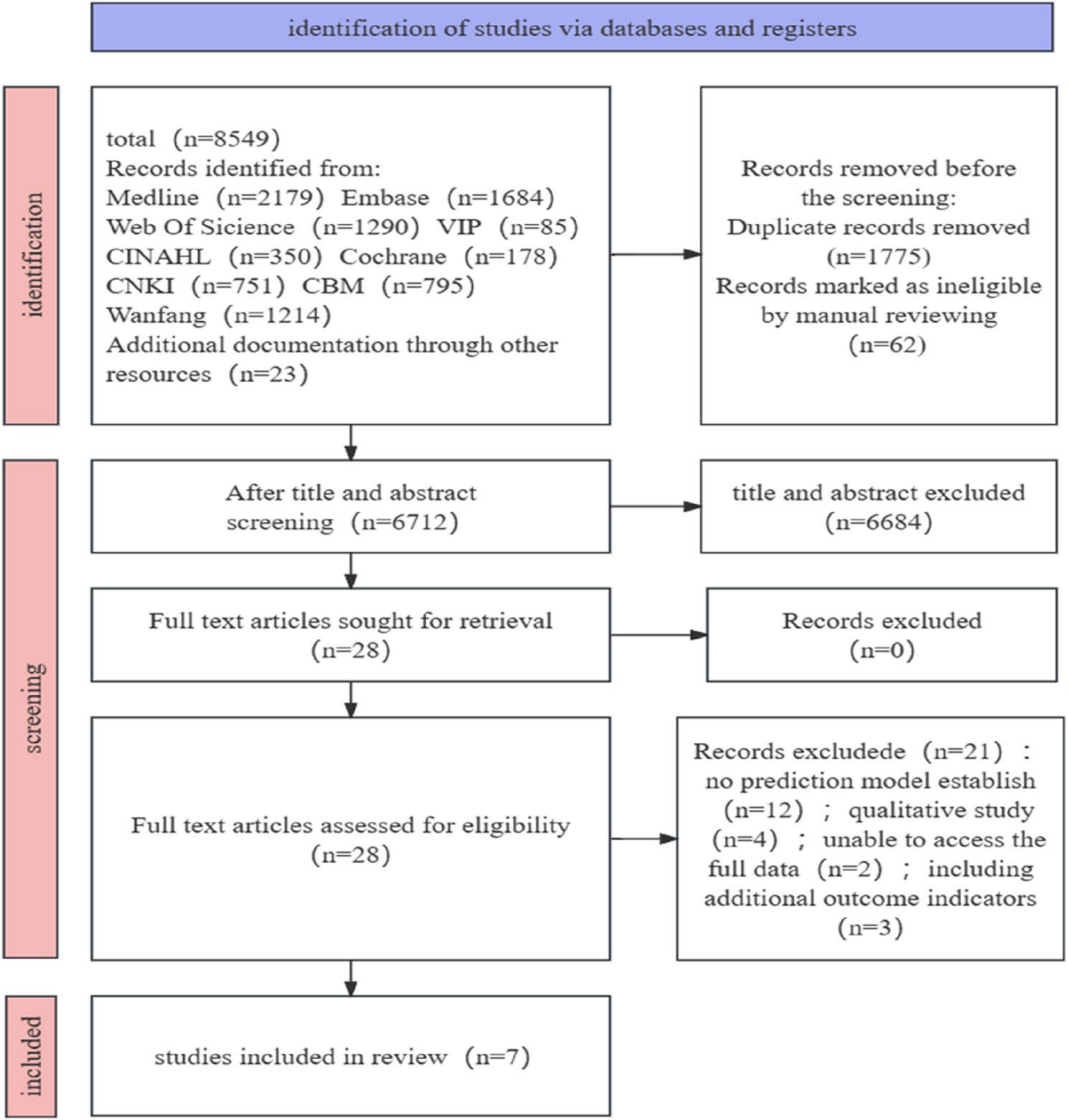
Flow chart of study selection

A total of 8549 studies were identified from nine databases and other sources. After removing 1775 duplicates and 62 records marking as ineligible by manual reviewing, 6172 articles remained. Through screening the title and abstract of the article, the remaining 28 records were reviewed by full-text screening, among which 12 records had not established prediction model, 4 records were qualitative study, 2 records were unable to access the full data, 3 records included additional outcome indicators. Finally, 7 articles were included in this study (Fig. 1).

### Study characteristics

7 studies yielded 19 models and published from 2020 to 2023, with four [24–26, 29] published in 2023. The 7 included models were from four different continents, including China (*n* = 4, 57.1%), Korea (*n* = 2, 28.6%), and Thailand (*n* = 1, 14.3%). Most of prediction models (*n* = 5, 71.4%) were developed using data from retrospective studies. Sample sizes included in the model were between 319 and 4417 cases, and patients with postmenopausal osteoporosis ranged from 157 to 837. Study characteristics were summarized in Table 1.

**Table 1:** Study characteristics (*n* = 7)

Study	Year	Country	Data source	Number of participants/number of events (EPV)
Shim JG et al [23]	2020	Korean	Retrospective study	1792/613 (EPV<20)
Wu Y et al [24]	2023	China	Retrospective study	4417/837 (EPV<20)
Wang SR et al [25]	2023	China	Case-control	323/185 (EPV<20)
Liu GK et al [26]	2023	China	Retrospective study	350/157 (EPV<20)
Kwon Y et al [27]	2022	Korean	Retrospective study	1431/821 (EPV<20)
Makond B et al [28]	2022	Thailand	Retrospective study	356/266 (EPV<20)
Wang JL et al [29]	2023	China	Case-control	319/159 (EPV<20)

### Model build and predictive performance

The number of predictors included in the final model predictors ranged from 3 to 20 and the main feature categories selected for building the predictive model were demographic characteristics (primarily including age, BMI, age at menopause, height, etc.), laboratory indicators (primarily biochemical and serum markers), other indicators (primarily lifestyle factors, medication use, and nutritional intake). The final inclusion of the predictors in the 7 models was detailed in Supplementary File 1 (Figure S3 Included predictors of each study). For predictor selection final to modeling, the most frequently applied method was logistic regression [23, 24, 26, 29]. As for the way to handle missing data, 3 studies [25, 26, 29] did not report missing values, other studies were imputed using multiple imputation or eliminated directly (Table 2).

**Table 2:** Model build and predictive performance (*n* = 7)

Study	Number of candidate predictors/predictors included in model	Type of predictors	Predictor selection	Missing data
Shim JG et al [23]	19/9	①	logistic regression (backward stepwise)	directly eliminate
Wu YQ et al [24]	17/9	②	multivariate logistic regression	eliminate and multiple imputation
Wang SR et al [25]	26/6	③	LASSO	not reported
Liu GK et al [26]	15/5	①	logistic regression	not reported
Kwon Y et al [27]	NR/20	③	recursive feature elimination	eliminate
Makond B et al [28]	NR/11	①	NR	K-nearest neighbor imputation
Wang JL et al [29]	6/3	①	logistic regression	not reported

*Abbreviations:* ①demographic characteristics, ②demographic characteristics and other indicators, ③demographic characteristics, laboratory indicators and others

*LASSO* Least Absolute Shrinkage and Selection Operator, *NR* Not Reported

As for model development, the logistic regression method was commonly used except for Makond B et al.’s [28] study, in which decision trees machine learning methods were used to generate prediction models, and Kwon Y et al. [27] applied other 3 machine learning methods (random forest, AdaBoost, gradient boosting machine). On the model validation, among the six studies [23–28] that employed internal validation of which 2 studies [27, 28] reported an 8:2 training-test split, 1 study [23] used a 7:3 split ratio. The remaining 3 studies [24–26] failed to specify their validation methodology. Only Wang JL et al.’s [29] study was externally validated using temporal validation. The model presentation format was reported in 4 models (57.1%), of which all models were presented as nomogram. Specific information provided in Table 3.

**Table 3:** Model build and predictive performance (*n* = 7)

Study	Model development methods	Type of validation	Model presentation
Shim JG et al [23]	LR, KNN, DT, RF, GBM, SVM, ANN	k-fold cross-validation	not reported
Wu YQ et al [24]	LR, Bayesian	sample splitting	Nomogram
Wang SR et al [25]	LR	internal validation	Nomogram
Liu GK et al [26]	LR	sample splitting	Nomogram
Kwon Y et al [27]	RF, AdaBoost, GBM	k-fold cross-validation	not reported
Makond B et al [28]	CART, QUEST, CHAID, C4.5	10-fold cross-validation	not reported
Wang JL et al [29]	LR	external validation	Nomogram

*Abbreviations:* *LR* Logistic Regressio, *KNN* K-Nearest Neighbors, *DT* Decision Tree, *RF* Random Forest, *GBM* Gradient Boosting Machine, *SVM* Support Vector Machines, *ANN* Artificial Neural Network, *CART* Classification and Regression Tree, *QUEST* Quick, Unbiased, Efficient Statistical Tree, *CHAID* Chi-squared Automatic Interaction Detection

In model validation, the reported C-index ranged from 0.639 to 0.921. Regarding model calibration performance, only three studies [24, 25, 29] provided both calibration curves and reported the results of the Hosmer-Lemeshow test (all p-values > 0.05), while one study [26] provided a calibration curve. For other performance metrics, four studies [23, 24, 26, 28] reported accuracy (ranging from 0.644 to 0.849), and four studies [23, 24, 28, 29] reported sensitivity (0.580 to 0.875) and specificity (0.560 to 0.880). Most studies [24–26, 28, 29] also reported metrics such as Youden’s index, positive predictive value, and negative predictive value, with some studies including decision curves (details are provided in Table 4).

**Table 4:** Model performance characteristics(*n* = 7)

Study	ML methods	Model performance				
		AUROC (95%CI)	Accuracy (95%CI)	Sensitivity (95%CI)	Specificity (95%CI)	Other indicators
Shim JG et al [23]	LR	0.727(0.672–0.753)	0.749(0.706–0.789)	0.79(0.72–0.85)	0.66(0.60–0.72)	-
	KNN	0.713(0.687–0.778)	0.747(0.704–0.787)	0.58(0.50–0.66)	0.85(0.80–0.89)	
	DT	0.685(0.641–0.731)	0.706(0.661–0.748)	0.60(0.52–0.68)	0.77(0.71–0.82)	
	RF	0.734(0.688–0.773)	0.747(0.704–0.787)	0.68(0.61–0.75)	0.79(0.73–0.83)	
	GBM	0.728(0.672–0.755)	0.718(0.673–0.759)	0.77(0.70–0.83)	0.69(0.63–0.74)	
	SVM	0.728(0.674–0.758)	0.727(0.682–0.768)	0.73(0.66–0.80)	0.72(0.67–0.78)	
	ANN	0.743(0.693–0.777)	0.749(0.706–0.789)	0.72(0.64–0.79)	0.77(0.71–0.82)	
Wu Y et al [24]	LR	0.752(0.734–0.768)	0.644	0.762	0.616	H-L (*P* = 0.125), calibration curve (slope value ≈ 1) Jorden index
	Bayesian	0.764(0.747–0.780)	0.678	0.708	0.672	
Wang SR et al [25]	LR	0.915(0.876–0.954)	-	-	-	H-L (*P* > 0.05), calibration curve decision curve
Liu GK et al [26]	LR	AUROC_1_= 0.792(0.745–0.840) AUROC_2_= 0.814(0.756–0.870)	-	-	-	calibration curve decision curve
Kwon Y et al [27]	RF	0.919	0.832	-	-	-
	AdaBoost	0.921	0.849			
	GBM	0.908	0.829			
Makond B et al [28]	CART	0.702	0.784	0.84	0.600	PPV = 0.815 NPV = 0.500
	QUEST	0.773	0.722	0.667	0.880	PPV = 0.842 NPV = 0.375
	CHAID	0.735	0.804	0.875	0.600	PPV = 0.828 NPV = 0.667
	C4.5	0.639	0.711	0.764	0.560	PPV = 0.808 NPV = 0.444
Wang JL et al [29]	LR	0.711(0.656–0.767)	0.742	0.581	-	calibration curve decision curve

*Abbreviations:* AUROC_1_, training model, AUROC_2_, testing model, *PPV* Positive Predictive value, *NPV* Negative predictive value, *HL* Hosmer-Lemeshow goodness-of-fit test

### Meta analysis

**Fig. 2:**
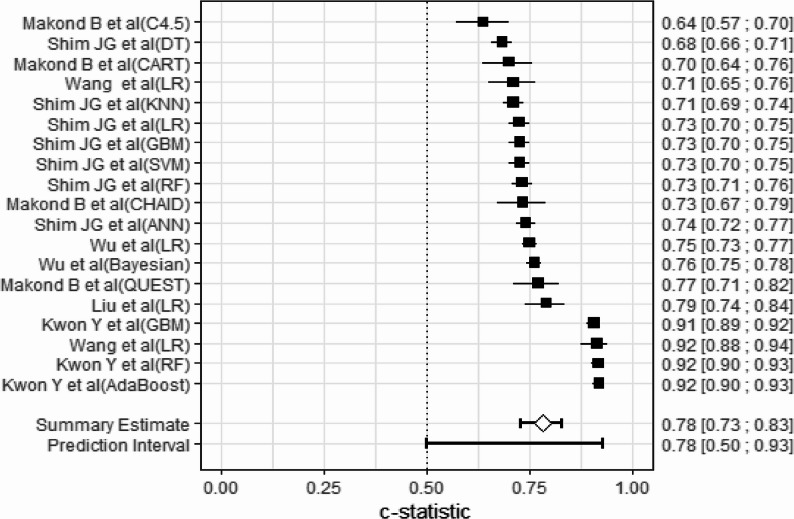
Forest plot of C statistics

We merged the C statistics of each model (Fig. 2). The results showed that the pooled C-statistic for the postmenopausal osteoporosis prediction model was 0.78 (95% CI: 0.73, 0.83). There was a high degree of heterogeneity among the included studies (I² = 98.0%, τ² = 0.0071), with a 95% prediction interval ranging from 0.54 to 0.93, which primarily attributable to variations in data sources, modeling approaches, and type of predictors. Therefore, we conducted meta regression and subgroup analyses to explore the sources of heterogeneity.

Meta-regression analysis revealed that the type of predictors was a significant source of performance heterogeneity (see Supplementary File 1, Table S4 meta-regression analysis of subgroups). Subgroup analysis results indicated that models incorporating demographic, laboratory, and other indicators reported the highest AUC value (0.92), and this difference was statistically significant. It was important to emphasize that this subgroup with the high AUC value included only 2 studies with a small sample size. Data source and modeling method were not significant sources of heterogeneity (Supplementary File 1, Table S5, subgroup analysis).

In the leave-one-out sensitivity analysis, the meta-analysis results showed an AUC = 0.77 (95% CI:0.73–0.81, *P* < 0.001) with heterogeneity I²=98.0%. Compared to the overall meta- analysis result of all models (AUC = 0.78, 95%CI: 0.73–0.83), the results demonstrated that the pooled AUC remained relatively stable upon individual study removal in sensitivity analyses. The random-effects meta-analysis results were provided in Supplementary File 1 (Figure S6 sensitive analysis results).

This study assessed publication bias through Egger’s regression test (*t* = −3.67, *P* = 0.0019) and trim-and-fill analysis. The Egger’s test revealed significant small-study effects (bias estimate = −9.14, SE = 2.49), indicating funnel plot asymmetry with smaller studies clustered in higher AUC regions (Supplementary File 1 Figure S7 funnel plot and egger’s regression test). Following trim-and-fill adjustment for 9 theoretically missing studies, the pooled AUC decreased from the original estimate of 0.845 to 0.795. Notably, extreme heterogeneity persisted throughout (I² = 98%, tau² = 0.0174) without significant improvement post-adjustment (Supplementary File 1 Figure S8 trim-and-fill adjustment).

### Risk of bias

**Fig. 3:**
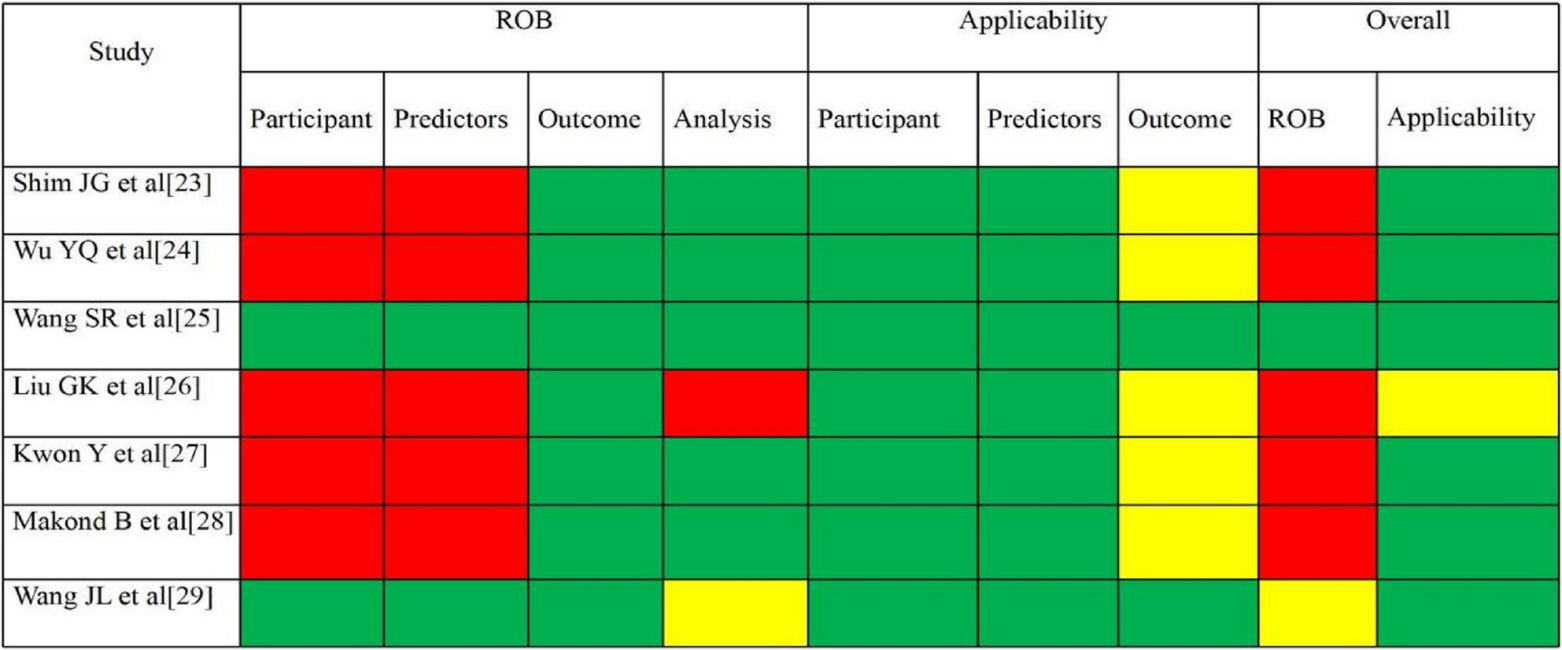
Risk of bias assessment. Abbreviations: Red:high risk; Yellow:unclear; Green:low risk [23–29]

A total of 7 studies were ultimately included, comprising 1 study with low risk of bias, 1 study with unclear risk of bias, and 5 studies with high risk of bias (Fig. 3). In the participants domain, the primary reason for high risk of bias was the retrospective study design (*n* = 5, 71.4%) [23, 24, 26–28], which might have introduced bias in case selection and control group definition. In the predictors domain, most of studies with high risk of bias (*n* = 5, 71.4%) [23, 24, 26–28] failed to report blinded assessment or standardized measurement methods for predictors and outcome indicators, potentially leading to measurement bias and expectation bias. In the analysis domain, the most common shortcomings were insufficient sample size (*n* = 7, 100%, EPV < 20) and risk of overfitting (*n* = 7, 100%). One additional study (*n* = 1, 14.3%) [26] was rated as high risk due to variable selection based solely on univariate analysis. This screening method could lead to the omission of important predictors and reduced model stability. The overall applicability of the 7 studies was better because the participants included were easy to find in the clinic and the relevant data of clinical diagnosis and treatment were also taken into account when selecting the variables.

## Discussion

This study conducted a literature search on risk prediction models for postmenopausal osteoporosis both domestically and internationally, ultimately including 7 studies with a total of 19 risk prediction models. All studies reported model discrimination (0.639–0.921), indicating that most models had moderate to excellent discrimination. However, three studies [23, 24, 27] failed to report calibration metrics, significantly limiting the evaluation of their clinical credibility. Calibration performance, which reflects the agreement between a model’s predicted probabilities and actual observed probabilities, serves as a critical indicator of a risk prediction model’s clinical utility [30]. The lack of calibration assessment may lead to several important issues. It prevents confirmation of whether the model’s predicted absolute risks are accurate - even with good discrimination, predicted probabilities may systematically overestimate or underestimate actual risks. It makes clinicians hesitant to trust the specific risk values provided by the model, thereby limiting its application in individualized decision-making [31, 32].

Furthermore, clinical utility represents the final critical step in evaluating the value of a prediction model. Among the 19 models included in this study, only three studies (encompassing 3 models in total) performed DCA to assess the models’ net benefit. DCA provides crucial evidence for practical application by quantifying the clinical benefit of using the model compared to default strategies (such as intervening on all or no patients) across various threshold probabilities [33]. The absence of DCA in the vast majority of studies means it is impossible to determine at which risk thresholds a model provides superior clinical value over simple strategies. This lack of evidence supporting the model’s ability to improve patient outcomes or enhance medical decision-making efficiency suggests that its so-called “high discriminative ability” may not translate into meaningful clinical net benefit. Similar to the lack of calibration assessment, the absence of clinical utility evaluation severely limits these models’ potential to evolve from mere statistical tools into reliable clinical decision support systems. Based on the above findings, we recommend that future developers of postmenopausal osteoporosis prediction models should be required to mandatorily report the model’s calibration performance (e.g., calibration plot, calibration slope) and clinical utility (e.g., DCA) to comprehensively demonstrate its clinical applicability. Furthermore, it is strongly recommended to strictly adhere to international guidelines such as PROBAST during the model development phase. This will help control the risk of bias across various aspects, including study design, data quality, analytical methods, and result reporting, thereby enhancing the model’s scientific rigor and generalizability potential.

Although external validation is crucial for assessing a model’s generalizability, this review found that only one study [27] performed external validation. This finding aligns with the results of Ramspek CL et al. [34], which indicated that only approximately 5–7% of prediction model studies complete external validation. This widespread lack of external validation creates significant uncertainty regarding the performance of numerous models in real-world clinical practice. It is particularly noteworthy that all prediction models included in this review originated from Asian countries, and the search strategy was limited to Chinese and English literature, potentially introducing geographical and language selection bias. This limitation implies that directly applying these models to non-Asian populations may lead to performance degradation or even clinical decision risks due to race-specific biological differences, variations in healthcare systems, or the failure of key predictors. Research confirms that ethnic and regional factors (e.g., genetic background, lifestyle) significantly influence the weighting of osteoporosis-related risk factors. A multi-center cohort study showed that East Asian women have a higher risk of hip fracture at the same bone mineral density compared to White women [35]. Another Mendelian randomization study found that although African Americans have a high prevalence of vitamin D deficiency, their incidence of osteoporotic fractures is relatively low, suggesting racial differences in the association between vitamin D and fracture risk [36]. The study by Lehmann O et al. [37] revealed that when a fracture prediction model developed based on a Swiss cohort was validated in the UK, the effectiveness of its key predictors (such as the number of falls) and the model’s performance both underwent significant changes. These differences affect not only the statistical performance of the models but also constrain their clinical applicability across diverse populations. It is important to clarify that this study focuses on risk prediction for postmenopausal osteoporosis itself, not fracture prediction. Given the current general lack of cross-ethnic validation frameworks for PMOP without fracture models, we strongly recommend exercising extreme caution when considering the application of the Asian models identified in this review to non-Asian populations.

In terms of model performance, the pooled AUC value in this study was 0.78 (95% CI: 0.73, 0.83), but significant heterogeneity (I² = 98%) was observed among the models. It is noteworthy that the sample size did not significantly affect the pooled AUC (*p* = 0.783). This may be because larger sample studies included more complex cases, leading to a reasonable performance decline, while smaller sample studies (EPV < 20) might have overestimated performance due to the risk of overfitting. The combined effects of small-study effects and publication bias may have led to an overestimation of the pooled AUC observed in this review and increased uncertainty in performance evaluation. Therefore, the pooled AUC should be interpreted as the average performance of the included studies rather than a generalizable single effect. Nevertheless, this meta-analysis provides a comprehensive overview of the current evidence and identifies factors that may influence performance (such as the type of predictors), thereby clarifying directions for future research. Currently, most of the included models rely on conventional clinical variables. However, with the rapid development of molecular techniques in recent years, serum biochemical markers have played an increasingly important role in the diagnosis and screening of osteoporosis. Studies have shown that changes in bone metabolism markers such as β-CrossLaps, N-MID osteocalcin, and PINP, along with their sensitivity, occur significantly earlier than changes in BMD [38, 39]. Therefore, developing prediction models that combine such bone turnover markers with BMD is a highly promising approach to enhance the accuracy of fracture-free PMOP risk prediction in the future. Nevertheless, it is important to note that the clinical translation of new predictors faces multiple obstacles, including measurement costs, accessibility, and assay turnaround time. Ultimately, prospective studies are still required to evaluate whether the application of these models genuinely improves patient outcomes or healthcare efficiency.

In terms of modeling algorithms, within the conditions of the included studies in this analysis, the performance of machine learning models did not significantly surpass that of logistic regression. This observation requires cautious interpretation, as it is influenced by several methodological limitations. First, many studies had small sample sizes and a limited number of events (EPV < 20), falling far short of the data scale required for machine learning models to fully demonstrate their advantages [40]. Second, carefully constructed features and the appropriate selection of predictors often contribute more to performance improvement than merely using complex algorithms [41]. The best-performing models (AUC > 0.9) in this analysis often integrated multiple types of important information (demographics and laboratory tests and others), which was likely the key to their high performance, rather than being solely attributable to a specific algorithm. Finally, the performance of machine learning models is highly dependent on hyperparameter tuning, which the included studies may not have adequately reported or optimized. This further complicates a fair comparison between ML and LR performance within the current analysis. Therefore, in small to medium-sized samples, logistic regression, with its simpler structure and clearer assumptions, may demonstrate more stable performance. Logistic regression holds enduring value in medical prediction modeling due to its interpretability, simplicity, and computational efficiency. Future research should more reliably evaluate the relative value of different algorithms in scenarios with sufficient data volume, complex features, and strong non-linear relationships. Model selection should be based on the specific problem, data characteristics, and the need for clinical interpretability, rather than blindly pursuing algorithmic complexity.

In this study, the predictive performance of the models was relatively good, but there were some methodological limitations in model development and validation, leading to a generally high risk of bias in the included studies. The primary causes of high risk of bias included overfitting risks, insufficient sample sizes, and inadequate handling of missing data. Among these, an insufficient sample size (EPV < 20) is the most direct cause of overfitting, which can significantly overestimate model performance metrics (such as AUC) and severely impact the model’s calibration and discriminative abilities in new populations. Regarding data sources, most of the data in this study were derived from databases or clinical records, utilizing retrospective data to construct the models. With the advancement of the information age, public databases have increasingly demonstrated their utility in the medical field due to their large sample sizes and strong statistical power. While such data are easily accessible and convenient, this design is prone to bias in establishing temporal relationships between predictors and outcomes [42], and interact with non-standardized measurements, further exacerbating model calibration errors and leading to systematic biases in risk prediction. Prospective cohort designs, randomized controlled trials, or well-designed nested case-control studies should be prioritized for future model development as they provide more robust evidence for establishing causal relationships. All studies had an events per variable (EPV) value of less than 20, likely contributing to its unstable validation results. Research shows EPV ≥ 20 for robust inference [20], underscoring the need for larger datasets in PMOP prediction. Future studies must establish stringent EPV targets a priori. When working with limited data, researchers should proactively employ feature selection techniques, parsimonious model architectures or enalized regression approaches to effectively manage model complexity while maintaining predictive validity. During the research implementation phase, some studies employed only univariate analysis to screen for predictive factors, which is extremely detrimental to the model. Not only can it lead to the omission of important predictors due to multicollinearity among independent variables, but it also significantly increases the probability of Type I errors. As a result, the final model may contain numerous spurious associations, substantially compromising its extrapolation performance [43]. Stepwise regression or penalty-/embedded-based feature selection methods demonstrate superior efficacy in handling multicollinearity and performing variable screening [44]. Deng F et al. [45] demonstrated in colorectal cancer research that an RFE-based feature selection framework incorporating multiple algorithms (logistic regression, SVM, random forest, XGBoost, and stacking) can effectively enhance classification performance when handling high-dimensional, redundant, and imbalanced data. In addition to statistical considerations, potential predictors should be comprehensively selected based on clinical relevance, measurement accessibility, and associated costs. Regarding missing data handling, three studies [25, 26, 29] failed to report missing values, while other studies employed either multiple imputation or direct elimination. Improper handling of missing data can introduce selection bias, particularly when the data is not missing completely at random. This can lead to biased estimates of model parameters. Simply deleting missing data may result in the retention of a significant number of outliers in the analytical dataset, thereby compromising predictive accuracy and calibration precision [46]. We recommend employing appropriate missing data handling methods such as multiple imputation or weighting techniques tailored to the type and mechanism, thereby minimizing potential bias. Overall, deficiencies in the analytical domain (such as insufficient sample size and overfitting) pose the most direct and severe threats to model reliability, as they directly impact the model’s internal validity. In contrast, biases in the participant and predictor domains primarily affect the model’s external validity and clinical applicability. Future research should prioritize ensuring adequate sample sizes, employing advanced statistical methods to prevent overfitting, and simultaneously minimizing foundational biases through prospective designs and standardized measurements whenever possible.

This study identified multiple risk prediction models for postmenopausal osteoporosis, yet significant publication bias was observed. Systematic omission of negative results (underperforming models) likely led to overestimated pooled AUC values. The interaction between high heterogeneity and bias substantially increased uncertainty in model performance evaluation. These findings reflected substantial methodological or population characteristic variations in current prediction model research, coupled with the preferential publication of small-sample positive results. Future studies should incorporate grey literature searches, conduct individual participant data meta-analyses to correct for bias, and prioritize clinically implemented models that have undergone rigorous external validation, thereby avoiding overreliance on potentially inflated pooled estimates. On the other hand, the model’s clinical implementation faces several challenges. First, model complexity and data requirements pose significant barriers, with some models incorporating 10–15 predictor variables and relying on laboratory indicators often unavailable in primary care settings. Second, inadequate workflow integration creates additional burdens as most models lack automated interfaces with electronic health record systems. Third, clinicians’ trust in the interpretability, reliability, and practical utility of complex models particularly “black-box” algorithms remains a pivotal determinant of adoption. Clinicians require clear understanding of a model’s decision-making logic particularly which key variables drive high-risk predictions to establish trust and guide personalized interventions. The lack of clear clinical guidance and insufficient integration of model outputs with existing diagnostic-therapeutic workflows further hinder their implementation. To facilitate clinical translation, we propose a stepped implementation strategy: (1) developing simplified tools (retaining 3–5 core clinical variables) to balance accuracy and practicality [47], ensure clear, intuitive result outputs and then seamlessly integrated into clinical decision nodes; (2) embedding models into clinical information systems or developing mobile applications for automated calculations; (3) establishing multidisciplinary teams to adapt models locally, creating tailored versions for different healthcare tiers and enhancing model interpretability by integrating explanation techniques like SHAP values and LIME or developing simplified rule sets, thereby making prediction logic transparent to address clinicians’ “black box” concerns and foster trust-building; (4) Conducting implementation research to evaluate real-world barriers, including usability, workflow impact, clinician and patient acceptance, as well as the actual effects of models on clinical outcomes such as fracture rates, treatment adherence, and cost-effectiveness [48]. Implementation studies should include dedicated evaluations of changes in physician trust levels and utilization of interpretability tools. Crucially, successful clinical integration requires concurrent healthcare provider training which key content should focus on transparently communicating the model’s principles, result interpretation guidelines, limitations, and individualized clinical integration pathways to directly address clinicians’ concerns and facilitate informed adoption. And then establishing continuous improvement mechanisms through regular outcome evaluations, ultimately enabling the transition from research tools to clinical decision support systems.

### Limitation

This study has several limitations. First, significant heterogeneity existed among the included studies, primarily manifested through substantial variations in sample sizes and a predominance of retrospective designs, which may affect the generalizability of conclusions. Second, all studies originated from Asian populations, limiting the applicability of findings to other ethnic groups. Third, most studies inadequately reported calibration metrics, hindering comprehensive evaluation of model calibration performance. The literature search was restricted to studies published after 2014 and included only Chinese and English publications, which may have introduced selection bias by omitting important non-Chinese/non-English studies and pre-2014 evidence on model validation. However, given this study’s focus on machine learning models (which rapidly advanced post-2014) and Asia’s status as the most active region for osteoporosis prediction research, the core conclusions are likely less affected by this bias. Future studies should conduct multi-center prospective studies, employ standardized reporting guidelines (e.g., TRIPOD statement) and conduct model validation in more diverse populations.

## Conclusion

Current machine learning-based prediction models for postmenopausal osteoporosis without fractures, as presented in the included studies, demonstrate good discriminative ability but are generally characterized by a high risk of bias, a notable lack of calibration performance evaluation, and insufficient validation of clinical utility. Furthermore, existing models are developed entirely on Asian population data, which limits their generalizability to other populations. Future research should focus on strictly adhering to prediction model research guidelines (such as PROBAST), enhancing the reporting of model calibration and clinical utility, and assessing model generalizability through external validation in multi-center studies encompassing diverse ethnicities and regions.

## Supplementary Information

Supplementary Material 1.

## Data Availability

No datasets were generated or analysed during the current study.
