# Supplementary material for: Risk prediction models for postmenopausal osteoporosis: a systematic review and meta-analysis study
Source: BMC Musculoskelet Disord. 2026 Jan 8;27:52. doi: 10.1186/s12891-025-09385-2 (PMC12829196; doi:10.1186/s12891-025-09385-2)
Supplement: Supplementary file 1 — Supplementary Material 1. [file 12891_2025_9385_MOESM1_ESM.docx]

S1 Search strategy

1. Medline

|  | Search line | Results |
| --- | --- | --- |
| 1 | ((((Postmenopause[MeSH Terms]) OR (postmenopausal women[Title/Abstract])) OR (postmenopausal[Title/Abstract])) OR (Postmenopausal Period[Title/Abstract])) OR (Post menopausal Period[Title/Abstract]) | 74074 |
| 2 | (((((osteoporosis[MeSH Terms]) OR (Osteoporosis, Postmenopausal[MeSH Terms])) OR (bone loss[Title/Abstract])) OR (Bone Mineral Density[Title/Abstract])) OR (BMD[Title/Abstract])) OR (bone metabolism[Title/Abstract]) | 135,966 |
| 3 | **machine learning OR Transfer Learning OR Deep learning OR Prediction model OR artificial intelligence OR random forest OR artificial neural network OR ANN OR Support vector machine OR SVM OR Gradient Boosting Machine OR GBM OR Nomogram OR XGboost OR Logistic OR Decision tree OR External validation OR Cox** | 4,325,483 |
| 4 | (validat$ OR predict$.ti. OR rules) OR (predict$ AND (outcomes$ OR risk$ OR model$)) OR ((history OR variable$ OR criteria OR scor$ OR characteristic$ OR finding$ OR factor$) AND (predict$ OR model$ OR decision$ OR identif$ OR prognos$)) OR (decision$ AND (model$ OR clinical$ OR logistic models/)) OR (prognostic AND (history OR variable$ OR criteria OR scor$ OR characteristic$ OR finding$ OR factor$ OR model$)) | 5,009,473 |
| 5 | #3 OR #4 | 7,739,191 |
| 6 | #1 AND #2 AND #5 AND #Filters: **in the last 10 years** | 2179 |

1. Web of science

|  | Search line | Results |
| --- | --- | --- |
| **1** | ((((TS=(Postmenopause)) OR TS=(postmenopausal women)) OR TS=(postmenopausal)) OR TS=(Postmenopausal Period)) OR TS=(Post menopausal Period) | 104,260 |
| **2** | ((((((TS=(Osteoporosis)) OR TS=(Osteoporoses)) OR TS=(Bone Loss)) OR TS=(Age-Related Bone Loss)) OR TS=(Bone Mineral Density)) OR TS=(BMD)) OR TS=(bone metabolism) | 258,153 |
| **3** | (((((((((((((((((TS=(machine learning)) OR TS=(Transfer Learning)) OR TS=( Deep learning)) OR TS=(Prediction model)) OR TS=(artificial intelligence)) OR TS=( random forest)) OR TS=(artificial neural network)) OR TS=(ANN)) OR TS=( Support vector machine)) OR TS=(SVM)) OR TS=( Gradient Boosting Machine)) OR TS=(GBM)) OR TS=(Nomogram )) OR TS=(XGboost )) OR TS=(Logistic)) OR TS=(Decision tree)) OR TS=( External validation)) OR TS=(Cox) | 2.820,773 |
| **4** | #1 AND #2 AND #3 AND #Filters: **in the last 10 years** | 1290 |

1. CINAHL

|  | Search line | Results |
| --- | --- | --- |
| 1 | SU Postmenopause OR SU postmenopausal women OR SU postmenopausal OR SU Postmenopausal Period OR SU Post menopausal Period | 12,248 |
| 2 | SU Osteoporosis OR SU Osteoporosis OR SU Bone Loss OR SU Age-Related Bone Loss OR SU Bone Mineral Density OR SU BMD OR SU bone metabolism | 31,448 |
| 3 | machine learning OR Transfer Learning OR Deep learning OR Prediction model OR artificial intelligence OR random forest OR artificial neural network OR ANN OR Support vector machine OR SVM OR Gradient Boosting Machine OR GBM OR Nomogram OR XGboost OR Logistic OR Decision tree OR External validation OR Cox | 855,231 |
| 4 | (validat$ OR predict$.ti. OR rules) OR (predict$ AND (outcomes$ OR risk$ OR model$)) OR ((history OR variable$ OR criteria OR scor$ OR characteristic$ OR finding$ OR factor$) AND (predict$ OR model$ OR decision$ OR identif$ OR prognos$)) OR (decision$ AND (model$ OR clinical$ OR logistic models/)) OR (prognostic AND (history OR variable$ OR criteria OR scor$ OR characteristic$ OR finding$ OR factor$ OR model$)) | 1,722,167 |
| 5 | S3 OR S4 | 2,299,412 |
| 6 | S1 AND S2 AND S5 AND last 10 years | 350 |

1. Cochrane

|  | Search line | Results |
| --- | --- | --- |
| 1 | （Postmenopause OR postmenopausal women OR postmenopausal OR Postmenopausal Period OR Post menopausal Period）ti.ab.kw | 24,641 |
| 2 | (Osteoporosis OR Osteoporoses OR Bone Loss OR Age-Related Bone Loss OR Bone Mineral Density OR BMD OR bone metabolism)ti.ab.kw | 30,247 |
| 3 | (machine learning OR Transfer Learning OR Deep learning OR Prediction model OR artificial intelligence OR random forest OR artificial neural network OR ANN OR Support vector machine OR SVM OR Gradient Boosting Machine OR GBM OR Nomogram OR XGboost OR Logistic OR Decision tree OR External validation OR Cox)ti.ab.kw | 89,499 |
| 4 | S1 AND S2 AND S3 AND last 10 years | 178 |

1. Embase

|  | Search line | Results |
| --- | --- | --- |
| 1 | 'postmenopause'/exp OR 'postmenopause' OR 'postmenopausal women'/exp OR 'postmenopausal women' OR 'postmenopausal' OR 'postmenopausal period'/exp OR 'postmenopausal period' OR 'post menopausal period' | 126,365 |
| 2 | 'osteoporosis' OR 'osteoporoses' OR 'bone loss' OR 'age-related bone loss' OR 'bone mineral density' OR 'bmd' OR 'bone metabolism' | 289,529 |
| 3 | 'machine learning' OR 'Transfer Learning' OR 'Deep learning' OR 'Prediction model' OR 'artificial intelligence' OR 'random forest' OR 'artificial neural network' OR 'ANN' OR 'Support vector machine' OR 'SVM' OR 'Gradient Boosting Machine' OR 'GBM' OR 'Nomogram' OR 'XGboost' OR 'Logistic' OR 'Decision tree' OR 'External validation' OR 'Cox' | 2,931,901 |
| 4 | S1 AND S2 AND S5 AND last 10 years | 1684 |

1. CNKI：

|  | Search line | Results |
| --- | --- | --- |
| 1 | SU=("绝经后妇女" +"绝经后" +"绝经" +"老年妇女") and SU=("骨质疏松症"+"骨量减少"+"骨量减少" +"骨密度" +"骨代谢") and SU=("深度学习"+"机器学习"+"人工智能"+"骨折风险评估工具"+"预测模型"+"列线图"+"模型"+"风险预测"+"风险评分"+"算法") | 751 |

7.Wanfang:

|  | Search line | Results |
| --- | --- | --- |
| 1 | 主题:("绝经后妇女" or "绝经后" or "绝经" or "老年妇女") and 主题:("骨质疏松症" or "骨量减少" or "骨量减少" or "骨密度" or "骨代谢") and 主题:("深度学习" or "机器学习" or "人工智能"or "骨折风险评估工具" or "预测模型" or "列线图" or "模型" or "风险预测" or "风险评分" or "算法") | 1214 |

8.VIP:

|  | Search line | Results |
| --- | --- | --- |
| 1 | M=(绝经后妇女 OR 绝经后 OR 绝经 OR 老年妇女) AND M=(骨质疏松症 OR 骨量减少 OR 骨量减少 OR 骨密度 OR 骨代谢) AND M=(深度学习 OR 机器学习 OR 人工智能 OR 骨折风险评估工具 OR 预测模型 OR 列线图 OR 模型 OR 风险预测 OR 风险评分 OR 算法) | 85 |

9.CBM

|  | Search line | Results |
| --- | --- | --- |
| 1 | ((深度学习 OR 机器学习 OR 人工智能 OR 骨折风险评估工具 OR 预测模型 OR 列线图 OR 模型 OR 风险预测 OR 风险评分 OR 算法) AND ("骨质疏松"[常用字段:智能] OR "骨量减少"[常用字段:智能] OR "骨密度"[常用字段:智能] OR "骨代谢"[常用字段:智能]) AND ("绝经后妇女"[常用字段:智能] OR "绝经后"[常用字段:智能] OR "绝经"[常用字段:智能] OR "老年妇女"[常用字段:智能])) AND 2014-2024[日期] | 795 |

**S2 Study Selection Criteria**

| **Inclusion Criteria** |
| --- |
| P **(Population):** postmenopausal women |
| I (**Index Models):** development, validation, or updating of prediction models for postmenopausal osteoporosis |
| C **(Comparator):**Not applicable |
| O (outcome):osteoporosis |
| S **(Study Design):**cohort study or case-control study |
| ****Exclusion Criteria**** |
| (1) the content of the study only referred to predictors or risk factors, but no predictive model had been established; |
| (2) used qualitative methods to construct prediction models; |
| (3) reported repeatedly, abstracts, conference papers, cases, and reviews; |
| (4) Non-Chinese and English. |

Figure S3 included predictors of each study


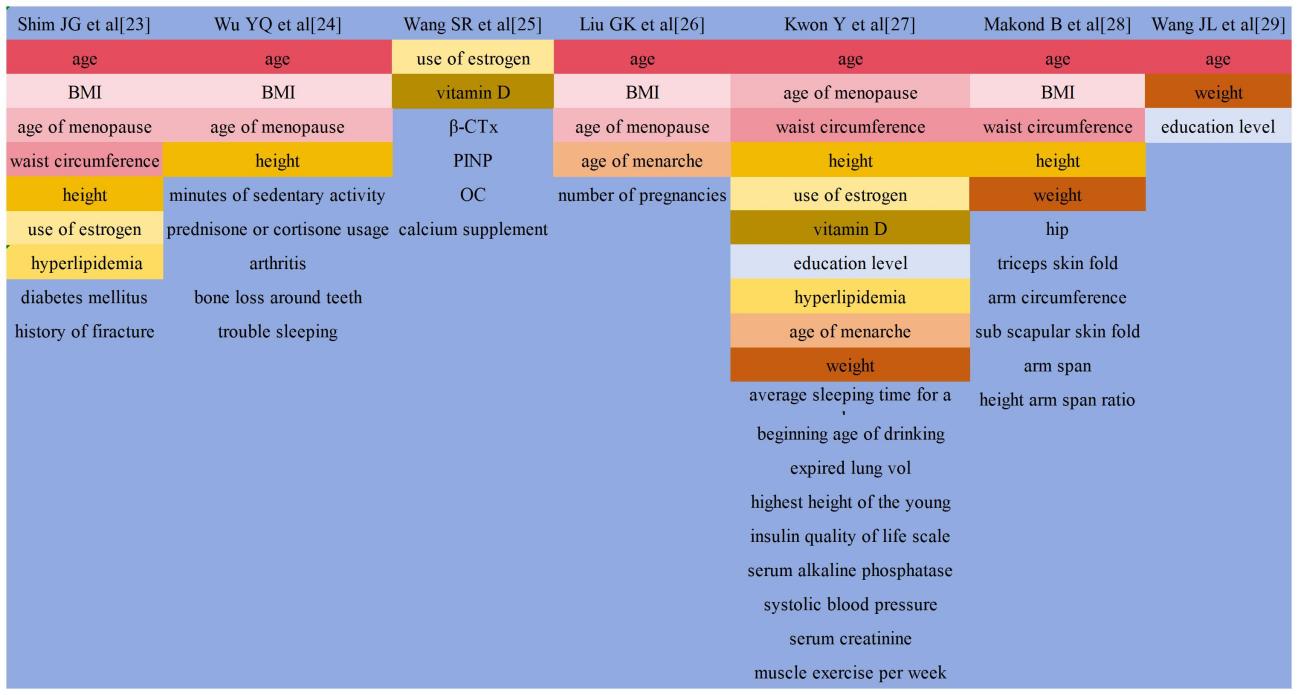


Table S4 meta regression analysis of subgroup

| Subgroup | Coef | SE | t | *P* value |
| --- | --- | --- | --- | --- |
| Data source | 0.004 | 0.014 | 0.275 | 0.783 |
| ML methods | -0.010 | 0.015 | -0.636 | 0.525 |
| Type of predictors |  |  |  |  |
| demographics and other indicators | 0.030 | 0.018 | 1.644 | 0.100 |
| demographics, laboratory and others | 0.193 | 0.015 | 13.051 | ＜0.0001 |

Table S5 subgroup analysis

| Subgroup | Number of predictive model | AUC（95%CI） | I^2^ value(%) | *P* value |
| --- | --- | --- | --- | --- |
| Data source |  |  |  |  |
| database | 12 | 0.78(0.73-0.83) | 98.4 | <0.0001 |
| electronic medical record | 7 | 0.75(0.70-0.81) | 92.6 | <0.0001 |
| ML methods |  |  |  |  |
| LR | 5 | 0.78(0.71-0.85) | 94.0 | <0.0001 |
| others | 14 | 0.77(0.72-0.81) | 98.2 | <0.0001 |
| Type of predictors |  |  |  |  |
| demographics | 13 | 0.72(0.70-0.74) | 59.3 | 0.003 |
| demographics and other indicators | 2 | 0.76(0.75-0.77) | 0.00 | 0.321 |
| demographics, laboratory and others | 4 | 0.92(0.91-0.92) | 0.00 | 0.625 |

Figure S6 sensitive analysis results


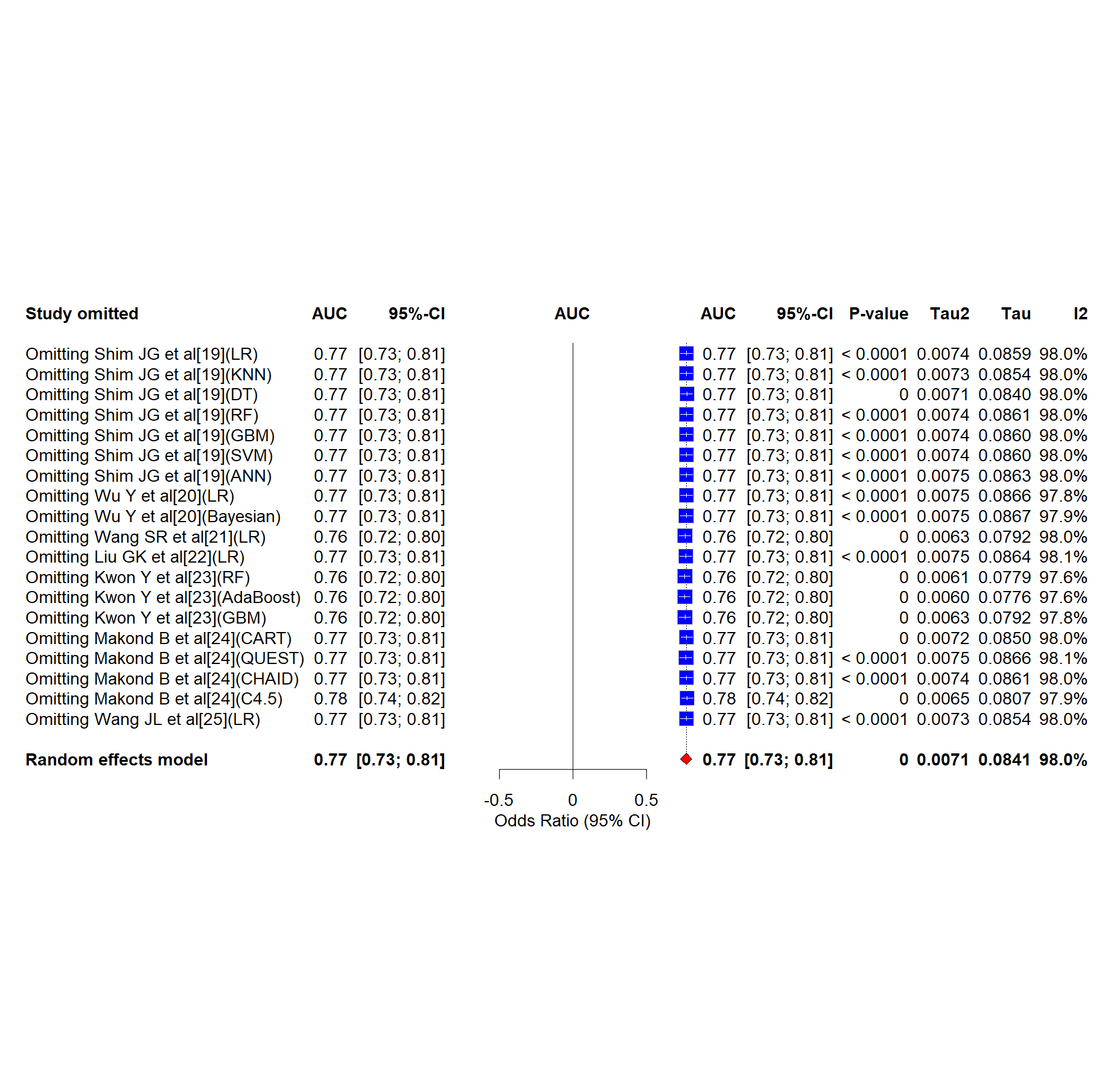


Figure S7 Funnel plot and egger's regression test

**
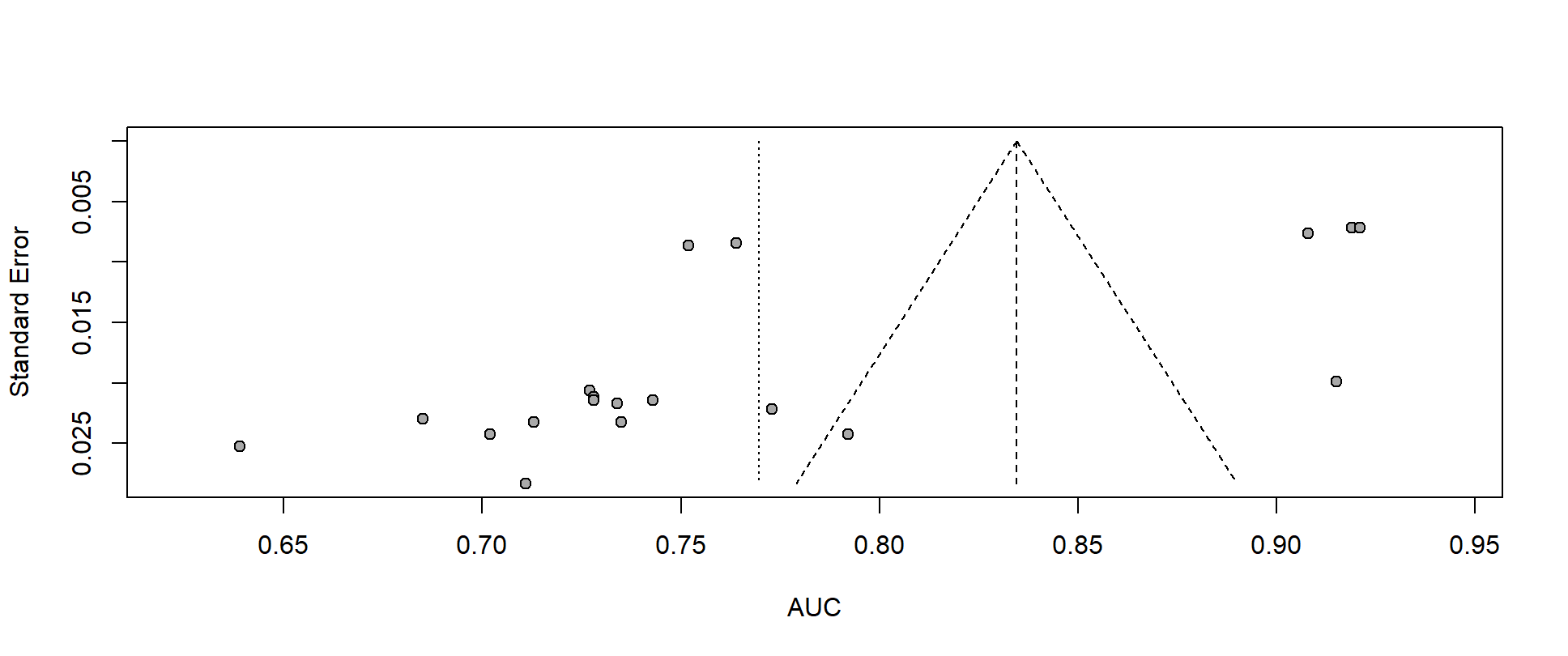
**

**
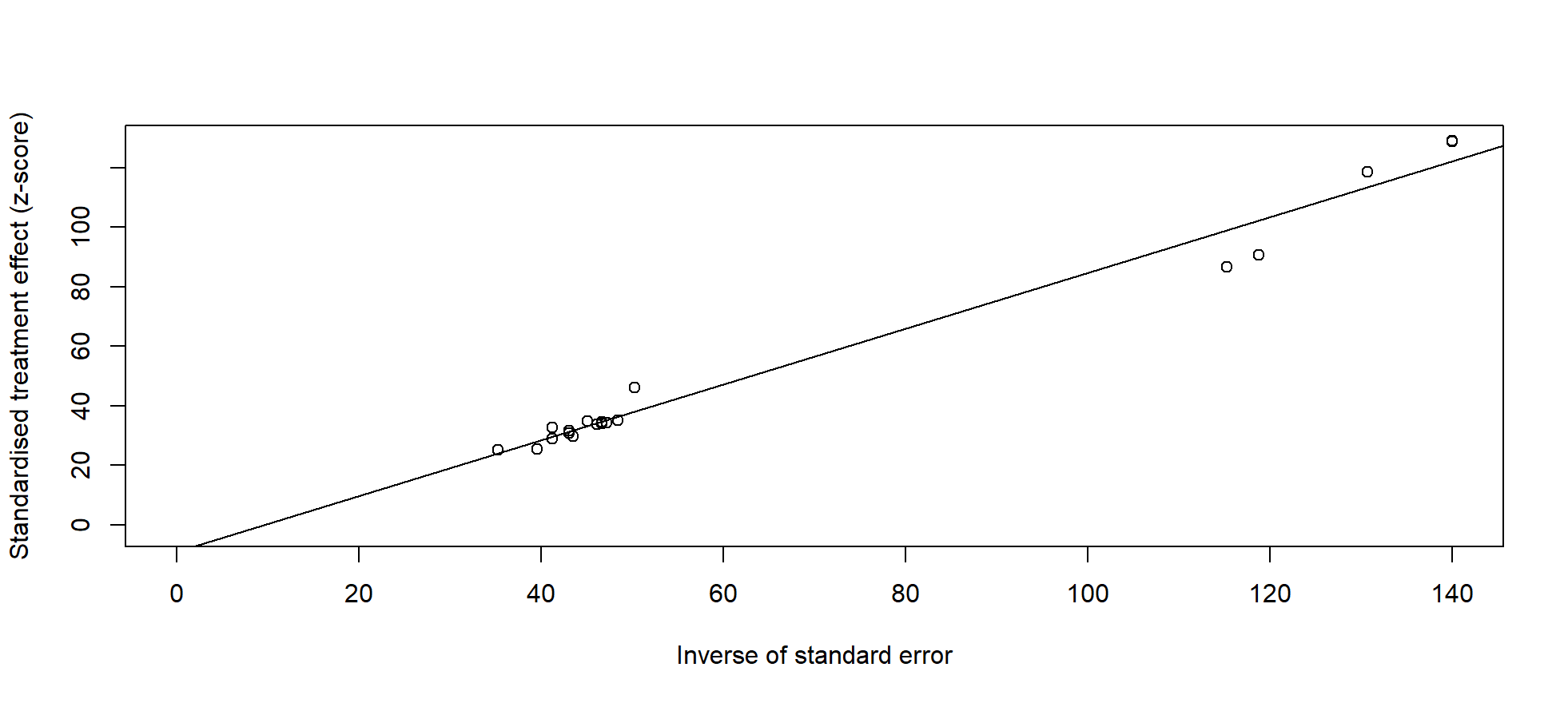
**

Figure S8 trim-and-fill adjustment

**
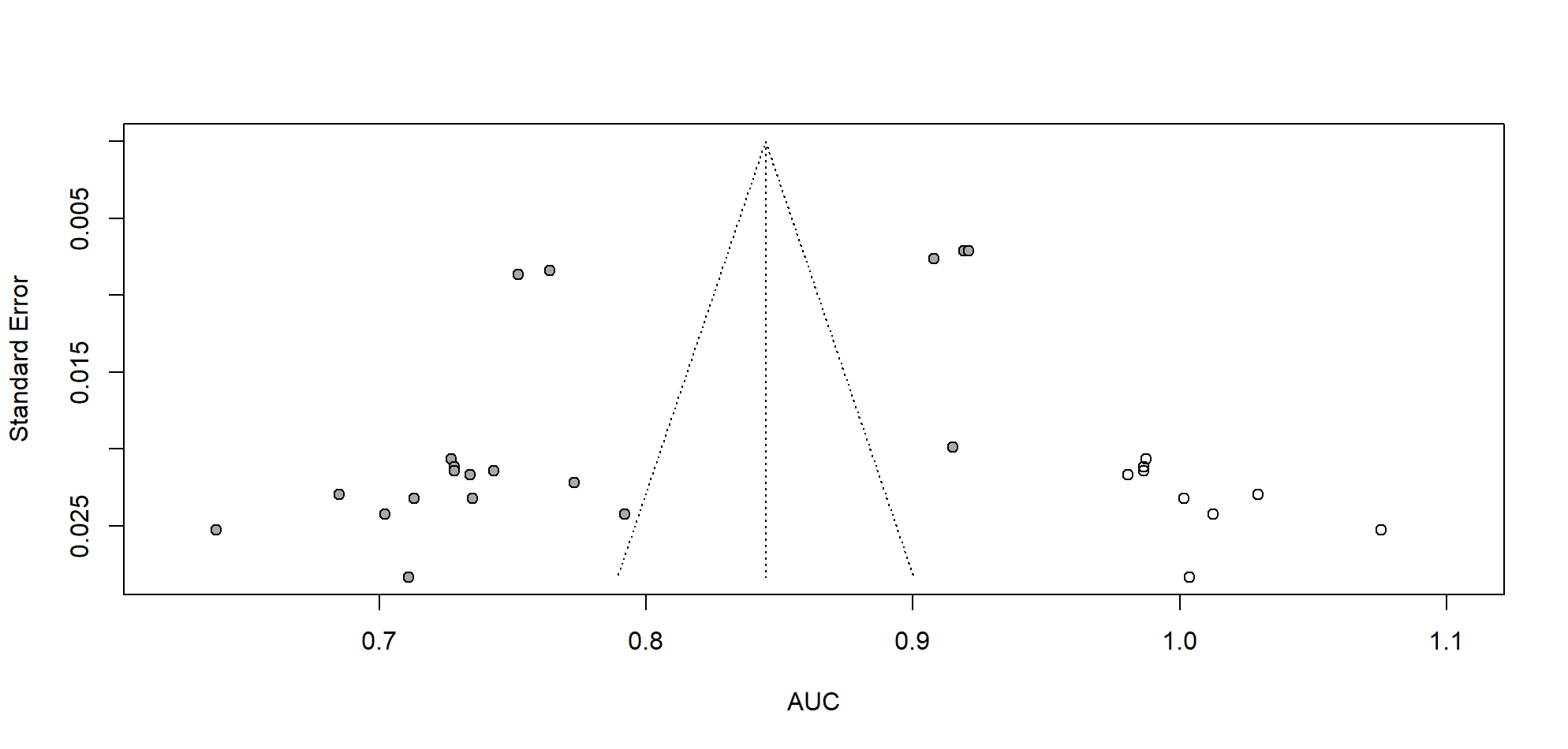
**
